# Declines in Sexual Activity and Function Predict Incident Health Problems in Older Adults: Prospective Findings from the English Longitudinal Study of Ageing

**DOI:** 10.1007/s10508-019-1443-4

**Published:** 2019-08-20

**Authors:** Sarah E. Jackson, Lin Yang, Ai Koyanagi, Brendon Stubbs, Nicola Veronese, Lee Smith

**Affiliations:** 1 Department of Behavioural Science and Health, University College London, 1-19 Torrington Place, London, WC1E 6BT UK; 2 Department of Epidemiology, Center for Public Health, Vienna, Austria; 3 Research and Development Unit, Parc Sanitari Sant Joan de Déu, Universitat de Barcelona, Fundació Sant Joan de Déu, Barcelona, Spain; 4 Instituto de Salud Carlos III, Centro de Investigación Biomédica en Red de Salud Mental, CIBERSAM, Madrid, Spain; 5 Physiotherapy Department, South London and Maudsley NHS Foundation Trust, Denmark Hill, London, UK; 6 Health Service and Population Research Department, Institute of Psychiatry, Psychology and Neuroscience, King’s College London, De Crespigny Park, London, UK; 7 Faculty of Health, Social Care and Education, Anglia Ruskin University, Chelmsford, UK; 8 Consiglio Nazionale delle Ricerche Area della Ricerca di Padova, Neuroscience Institut, Padua, Italy; 9 The Cambridge Centre for Sport and Exercise Sciences, Anglia Ruskin University, Cambridge, UK

**Keywords:** Sexual function, Sexual activity, Erectile dysfunction, Older adults, Health outcomes

## Abstract

The objective of this study was to investigate cross-sectional and longitudinal associations between declines in sexual activity and function and health outcomes in a large population-based sample of older adults. Data were from 2577 men and 3195 women aged ≥ 50 years participating in the English Longitudinal Study of Ageing. Past-year changes in sexual desire, frequency of sexual activity, and ability to have an erection (men)/become sexually aroused (women) were assessed at baseline by self-completion questionnaire. Health outcomes (self-rated health, limiting long-standing illness, doctor-diagnosed diseases of the vascular system, and cancer) were self-reported at baseline (2012/2013) and 4-year follow-up (2016/2017). Data were analyzed using logistic regression, adjusted for sociodemographics, health behaviors, and depressive symptoms. Prospectively, men who reported a decline in sexual desire had higher odds of incident limiting long-standing illness (OR 1.41, 95% CI 1.04–1.91) and incident cancer (OR 1.63, 95% CI 1.06–2.50) than those who maintained their sexual desire. Men who reported a decline in the frequency of sexual activities had higher odds of deterioration in self-rated health (OR 1.47, 95% CI 1.04–2.08) and incident limiting long-standing illness (OR 1.69, 95% CI 1.20–2.37). In women, a decline in frequency of sexual activities was associated with deterioration of self-rated health (OR 1.64, 95% CI 1.07–2.51). Erectile dysfunction was longitudinally associated with poorer health outcomes including incident cancer (OR 1.73, 95% CI 1.11–2.70), coronary heart disease (OR 2.29, 95% CI 1.29–4.07), and fair/poor self-rated health (OR 1.66, 95% CI 1.19–2.32). Practitioners should be mindful that a decline in sexual activity, desire, or function in older age may be an important indicator of future adverse health outcomes.

**Electronic supplementary material:**

The online version of this article (10.1007/s10508-019-1443-4) contains supplementary material, which is available to authorized users.

## Introduction

In high-income countries, life expectancy has risen considerably. For example, in the U.S. a boy born in 1900 was expected to live to 46.3 years and a girl to 48.3 years (Centers for Disease Control and Prevention, 2010); by 2016, the respective life expectancies were 76.3 years and 81.2 years (World Bank, 2018). However, as life expectancy has increased, there has also been a concurrent rise in years lived with disability and adverse health outcomes in older age (Crowther, Parker, Achenbaum, Larimore, & Koenig, 2002; Rowe & Kahn, 1997, 2015). As such, understanding factors that may contribute to or be an early warning sign of poor health is essential.

One key behavior that may influence health in later life but has received little attention in the literature is sexual activity. There is a common misconception that people become asexual as they get older, losing their interest in sex, and capacity for sexual behavior (DeLamater, 2012). On the contrary, while sexual activity does tend to decline with age, it remains a prevalent behavior. In a UK survey, the proportion of men who reported being sexually active was 84.5% in those aged 60–69 years, 59.3% in those aged 70–79 years, and 31.1% in those aged ≥ 80 years; in women, the respective prevalence was 59.9%, 34.3%, and 14.2% (Lee, Nazroo, O’Connor, Blake, & Pendleton, 2016). Similar levels have been reported in the U.S. population, where 73% of people aged 57–64 years, 53% of those aged 65–74 years, and 26% of those aged 75–85 years surveyed reported being sexually active (Lindau et al., 2007).

A handful of studies have indicated that an active and trouble-free sex life may be associated with reduced risk of disease outcomes among older adults. To our knowledge, the largest study thus far was of 1046 men and 1158 women (aged 57–85 years) residing in the U.S., with a 5-year follow-up. Results indicated that the frequency and quality of sex protected against cardiovascular events in later life (Liu, Waite, Shen, & Wang, 2016). Frequency of sexual intercourse has also been shown to be associated with reduced risk of fatal coronary events, and prostate and breast cancer in studies using a longitudinal or case–control design (Ebrahim et al., 2002; Le, Bachelot, & Hill, 1989). The apparent protective role of sexual activity for health may, at least in part, be attributable to the release of endorphins during sexual activity. Endorphin levels are associated with higher natural killer cell activity (Darko, Irwin, Risch, & Gillin, 1992) which is associated with a lower risk of several health complications, including cancer (Mandal & Viswanathan, 2015; Wu & Lanier, 2003). Moreover, sexual activity is a form of physical activity (Frappier, Toupin, Levy, Aubertin-Leheudre, & Karelis, 2013) and physical activity is associated with positive mental and physical health (McPhee et al., 2016; Sodergren, Sundquist, Johansson, & Sundquist, 2008). However, it is also possible that these results reflect people with better health being more able or inclined to engage in sexual activity than those with poor health.

While this evidence points to sexual activity as a potentially important predictor of health in older age, much of the research published to date is limited either by being theoretical (Bosland, 1988), cross sectional (Brody & Preut, 2003; Flynn & Gow, 2015), only considering a single health outcome, or consisting of a small sample size (Ebrahim et al., 2002; Le et al., 1989). These limitations make it difficult to draw any firm conclusions about any protective effects of sexual activity for health. Importantly, no studies have examined the extent to which changes in sexual well-being are predictive of health outcomes. Should such associations be established, asking older adults about any problems with their sex lives could be a simple way to identify those at risk of adverse health outcomes.

The aim of the present study was therefore to investigate the cross-sectional and prospective associations between changes in sexual activity and function and physical health outcomes in a large, probability-based sample of older adults. Specifically, we analyzed associations between declines in sexual desire, frequency of sexual activities, and ability to have an erection (men) or become sexually aroused (women), and self-rated health, limiting long-standing illness, cancer, coronary heart disease (CHD), and stroke over a four-year follow-up, in order to address the following research questions:

1. Cross sectionally among all older adults, are the odds of ill health higher among those who experience a decline in sexual desire, activity or function compared with those whose sexuality remains stable or increases?
2. Prospectively among older adults who are healthy at baseline, is a decline in sexual desire, activity or function associated with increased odds of incident illness over four-year follow-up?

## Method

### Participants

This study used data from the English Longitudinal Study of Ageing (ELSA) covering a four-year period (2012/13–2016/2017). ELSA is a population-based longitudinal panel study of men and women aged 50 and older living in England (Steptoe, Breeze, Banks, & Nazroo, 2013). The study started in 2002 (Wave 1), with participants recruited from an annual cross‐sectional survey of households (the Health Survey for England). The study was described to potential participants as a study “to explore the health, lifestyles, and financial situation of people as they grow older.” Participants are followed up every 2 years. For the present study, baseline data were from Wave 6 (2012/2013; the first wave in which sexual relationships and activities were assessed) and follow-up data were from Wave 8 (2016/2017; the latest wave for which data were available). The primary form of data collection in ELSA is a computer-assisted personal interview (CAPI) conducted face to face in the participant’s home or residence. Further data are obtained from self-completion questionnaires that respondents return to the research office by post after the CAPI.

There were 10,601 individuals interviewed in Wave 6 of ELSA, of whom 7079 (67% of those eligible) completed the paper‐based Sexual Relationships and Activities Questionnaire (SRA-Q). We restricted our sample to men and women who had data on at least one outcome variable at baseline and had complete data on covariates (*n *= 5772, 81.5%). Prospective analyses were performed on a subsample with data on at least one outcome variable at follow-up (*n *= 4686, 81.2% of the baseline sample). All participants gave full informed consent to participate in the study, and ethical approval was obtained from the London Multi‐Centre Research Ethics Committee.

### Measures

#### Measurement of Exposures: Changes in Sexual Activity and Function

Data on changes in sexual activity and function were collected via the SRA-Q, a questionnaire derived from previously validated measures with modifications to ensure comparability with the National Social Life, Health, and Aging Study in the U.S. (Suzman, 2009) and with the National Survey of Sexual Attitudes and Lifestyles in the UK (Field et al., 2013). The male and female versions of the SRA-Q are available online at http://www.elsa-project.ac.uk/documentation. Participants were advised that some questions may be of a sensitive nature, but that their answers would help in understanding possible changes in sexual relationships and activities as people grow older, the reasons for this, and how changes in sexual relationships relate to other aspects of people’s lives.

Participants were asked whether they had had any sexual activity (defined as sexual intercourse, masturbation, petting, or fondling) in the past year (yes/no). They were also asked whether, compared with a year ago, they had experienced a change in their (1) sexual drive/desire (all men and women), (2) overall frequency of sexual activities (sexually active men and women), and (3) ability to have an erection (all men)/become sexually aroused (sexually active women). Response options were “increased a lot,” “increased moderately,” “neither increased nor decreased,” “decreased moderately,” “decreased a lot.” We dichotomized responses to distinguish between participants reporting a moderate or large decrease in sexual desire, frequency, or function from those who reported no change or an increase (Lee et al., 2016).

#### Measurement of Health Outcomes

Self‐rated health was assessed using a single item: “Would you say your health is… poor/fair/good/very good/excellent?” We analyzed the proportion of individuals rating their health as fair/poor, as has been done in other investigations (e.g., DeSalvo, Bloser, Reynolds, He, & Muntner, 2006; Steptoe & Jackson, 2018). Limiting long-standing illness was self-reported in response to two questions: (1) “Do you have any long-standing illness, disability, or infirmity? By long-standing I mean anything that has troubled you over a period of time or that is likely to affect you over a period of time.” If yes, (2) “Does this illness or disability limit your activities in any way?” Affirmation of a long-standing illness and any form of limitation classified the participant as having a limiting long-standing illness. This measure captures any ongoing health condition that the participant perceives to have an ongoing impact on their life and is therefore a useful complement to specific diagnosed conditions. Information about doctor-diagnosed cancer, CHD, and stroke was self-reported in response to presentation of a list of conditions and asking: “Has a doctor ever told you that you have (or have had) any of the conditions on this card.”

#### Measurement of Potential Confounders

All potential confounders were selected a priori and assessed at baseline. Demographic information collected included age, sex, ethnicity (white vs. non-white) and partnership status (married/cohabiting, separated/divorced, widowed, or single/never married). Socioeconomic status (SES) was based on household non-pension wealth, an indicator that has been identified as particularly relevant to health outcomes in this age group (Banks, Karlsen, & Oldfield, 2003), categorized into quintiles across all ELSA participants who took part in Wave 6. Health-related questions included self-reported current smoking status (smoker or non-smoker) and frequency of alcohol intake, categorized as never/rarely (never—once or twice a year), regularly (once every couple of months—twice a week), or frequently (3 days a week—almost every day) (Lee et al., 2016). Physical activity was assessed with three items that asked participants how often they took part in vigorous, moderate- and low-intensity activities (more than once a week, once a week, 1–3 times a month, hardly ever/never) (Demakakos, Hamer, Stamatakis, & Steptoe, 2010). Physical activity was further categorized into three categories, as previously described (Hamer, Molloy, de Oliveira, & Demakakos, 2009): inactive (no moderate/vigorous activity on a weekly basis); moderate activity at least once a week; and vigorous activity at least once a week. Depressive symptoms were assessed using the eight-item Centre for Epidemiological Studies Depression (CES-D) scale, a validated scale for use in older adults (Cosco, Prina, Stubbs, & Wu, 2017; Steffick, 2000).

### Statistical Analysis

Analyses were performed using IBM SPSS Statistics 22. Data were weighted to correct for sampling probabilities and for differential non-response and to calibrate back to the 2011 National Census population distributions for age and sex. The weights accounted for the differential probability of being included in Wave 6 of ELSA and for non-response to the SRA‐Q. Details can be found at http://doc.ukdataservice.ac.uk/doc/5050/mrdoc/pdf/5050_elsa_w6_technical_report_v1.pdf. Cases with missing data were excluded on a per-analysis basis.

Characteristics of participants who did and did not provide follow-up data were compared using independent t-tests (continuous variables) and chi-square tests (categorical variables). We used logistic regression to test cross-sectional associations between decline in sexual desire, frequency and function and baseline health status, and prospective associations between decline in sexual desire, frequency and function and incident ill health (development of adverse health outcomes at follow-up in those who were healthy at baseline) over 4 years, adjusting for baseline age, partnership status, ethnicity, wealth, smoking status, alcohol intake, physical activity and depressive symptoms. Separate analyses were carried out on men and women. For each outcome, we report the odds ratio (OR) and 95% confidence interval (CI) for those who reported a decline in sexual desire, frequency or function, relative to those who did not report a decline.

In a sensitivity analysis, we explored the influence of excluding cases with missing data. A total of 1,283 participants who returned the SRA-Q were excluded for having missing data. The variables accounting for the majority of missing cases were wealth (missing for *n *= 963, 75.1% of excluded participants), alcohol (*n *= 173, 13.5%), sexual desire (*n *= 84, 6.5%), age (*n *= 62, 4.8%) and depressive symptoms (*n *= 55, 4.3%). Missing values for all relevant variables were multiply imputed using an imputation model (following the MCMC method (IBM, 2014)) with all other variables as predictors. Five imputed datasets were created, each analyzed separately, and the results combined to produce pooled estimates of effects, allowing the analyses to account for uncertainty caused by estimating missing data. Pooled estimates are reported in Supplementary Tables 1 and 2.

## Results

A total of 2577 men and 3195 women were included in the present analyses. Sample characteristics at baseline are given in Tables 1 (men) and 2 (women). The mean age of participants was 64.9 (SD 10.01) years. The majority were married or cohabiting, of white ethnicity, non-smokers, and regular or frequent drinkers. Of this overall sample, 2074 men and 2612 women provided follow-up data and were included in the prospective analyses.

**Table 1:** Sample characteristics: Men

	All men	Decline in sexual desire		Decline in frequency of sexual activities		Decline in ability to have an erection	
	(*n *= 2577)	Yes (*n *= 852)	No (*n *= 1725)	Yes (*n *= 732)	No (*n *= 1238)	Yes (*n *= 705)	No (*n *= 1850)
Age (mean [SD] years)	64.31 (9.75)	66.82 (10.14)	63.11 (9.33)	62.37 (8.67)	61.63 (8.10)	67.56 (9.99)	63.02 (9.30)
Partner status							
Married/cohabiting	73.7	78.4	71.4	78.4	74.2	77.6	72.5
Separated/divorced	11.5	8.5	12.9	11.0	12.8	7.8	12.9
Widowed	6.1	6.0	6.2	3.8	3.7	7.9	5.2
Single/never married	8.7	7.2	9.5	6.8	9.3	6.7	9.4
Ethnicity							
White	93.8	93.8	93.9	92.6	94.3	92.8	94.1
Non-white	6.2	6.2	6.1	7.4	5.7	7.2	5.9
Wealth quintile							
1 (poorest)	17.4	18.6	16.8	15.8	15.5	18.7	16.7
2	19.1	21.0	18.2	19.0	17.9	19.7	18.6
3	19.7	22.3	18.5	20.6	18.2	22.2	18.8
4	22.0	18.9	23.4	20.9	24.0	19.3	23.0
5 (richest)	21.8	19.2	23.1	23.7	24.3	20.1	22.7
Smoking status							
Non-smoker	85.5	85.6	85.5	83.9	86.1	86.7	85.1
Smoker	14.5	14.4	14.5	16.1	13.9	13.3	14.9
Alcohol intake^a^							
Never/rarely	16.0	16.4	15.8	10.8	13.6	16.4	15.6
Regularly	41.9	43.7	41.0	44.5	41.0	42.4	42.0
Frequently	42.1	39.9	43.2	44.7	45.4	41.2	42.4
Physical activity							
Inactive	19.7	23.7	17.7	17.5	13.7	24.2	17.9
Moderately active ≥ once a week	43.4	45.6	42.3	47.6	41.1	46.0	42.5
Vigorously active ≥ once a week	36.9	30.7	39.9	34.9	45.2	29.9	39.6
Depressive symptoms (0–8) (mean [SD])	1.13 (1.82)	1.33 (1.90)	1.04 (1.78)	1.28 (1.99)	0.89 (1.61)	1.54 (2.11)	0.97 (1.68)

Values are percentages unless otherwise stated

All figures are weighted for sampling probabilities and differential non-response

*SD* standard deviation

^a^Never/rarely = never—once or twice a year; regularly = once every couple of months—twice a week; frequently = 3 days a week—almost every day

**Table 2:** Sample characteristics: Women

	All women	Decline in sexual desire		Decline in frequency of sexual activities		Decline in ability to become sexually aroused	
	(*n* = 3195)	Yes (*n* = 1009)	No (*n* = 2186)	Yes (*n* = 663)	No (*n* = 1072)	Yes (*n* = 362)	No (*n* = 1004)
Age (mean [SD] years)	65.26 (10.07)	65.98 (10.55)	64.91 (9.81)	62.00 (8.64)	61.18 (7.93)	61.44 (8.30)	60.50 (7.77)
Partner status							
Married/cohabiting	61.6	68.2	58.4	80.1	75.8	80.5	75.5
Separated/divorced	15.8	12.3	17.5	12.0	13.9	12.2	14.8
Widowed	16.8	15.5	17.4	3.8	5.5	2.9	5.0
Single/never married	5.8	3.9	6.8	4.1	4.8	4.4	4.7
Ethnicity							
White	96.0	96.1	95.9	97.6	96.7	97.7	97.1
Non-white	4.0	3.9	4.1	2.4	3.3	2.3	2.9
Wealth quintile							
1 (poorest)	19.7	20.7	19.1	14.2	12.0	14.8	11.7
2	20.7	22.8	19.6	17.7	19.0	17.7	17.7
3	21.0	19.7	21.7	20.4	20.5	17.7	21.5
4	19.7	19.7	19.7	24.4	22.1	24.1	24.3
5 (richest)	18.9	17.1	19.8	23.3	26.5	25.6	24.8
Smoking status							
Non-smoker	86.6	85.0	87.4	90.3	85.5	85.2	87.0
Smoker	13.4	15.0	12.6	9.7	14.5	14.8	13.0
Alcohol intake^a^							
Never/rarely	29.7	31.7	28.8	22.7	19.5	19.5	19.7
Regularly	43.7	42.4	44.2	45.5	47.4	45.6	47.5
Frequently	26.6	25.9	27.0	31.9	33.2	34.9	32.8
Physical activity							
Inactive	25.1	28.6	23.5	17.2	16.4	16.6	15.6
Moderately active ≥ once a week	48.2	47.2	48.7	49.8	48.7	48.0	50.3
Vigorously active ≥ once a week	26.6	24.3	27.8	32.9	34.9	35.5	34.1
Depressive symptoms (0–8) (mean [SD])	1.56 (2.01)	1.76 (2.11)	1.47 (1.95)	1.62 (2.04)	1.16 (1.73	1.58 (2.08)	1.19 (1.74)

Values are percentages unless otherwise stated

All figures are weighted for sampling probabilities and differential non-response

*SD* standard deviation

^a^Never/rarely = never—once or twice a year; regularly = once every couple of months—twice a week; frequently = 3 days a week—almost every day

Compared with those who were not included, participants who were included in the prospective analyses tended to be more socio-demographically advantaged and in better health. They were significantly younger (mean [SD] 64.4 [9.4] vs. 66.3 [11.5] years, *p* < .001) and wealthier (% in top quintile 21.6% vs. 15.4%, *p* < .001) at baseline, and fewer were widowed (10.7% vs. 15.3%, *p* < .001) or from ethnic minority groups (4.6% vs. 6.8%, *p* = .002). Fewer were smokers (12.6% vs. 18.7%, *p* < .001) or non-drinkers (21.7% vs. 28.6%, *p* < .001). They reported fewer depressive symptoms (1.58 [2.06] vs. 1.30 [1.89], *p* < .001) at baseline and were less likely to rate their health as fair or poor (24.0% vs. 32.4%, *p* < .001) or report a limiting long-standing illness (32.3% vs. 38.3%, *p* < .001). In addition, a greater proportion of the sample retained for prospective analyses reported a decline in sexual desire (31.5% vs. 36.7%, *p* = .001), but there was no significant difference in the proportion reporting a decline in the frequency of sexual activity, ability to have an erection or ability to become sexually aroused (*p* > .05).

Cross-sectional and prospective associations between past-year decline in sexual desire, frequency of sexual activities, and sexual function and health status, adjusted for socio-demographic and health-related covariates, are given in Tables 3 (men, cross sectional), 4 (men, prospective), 5 (women, cross sectional) and 6 (women, prospective).

**Table 3:** Cross-sectional associations in men between past-year decline in sexual desire, frequency of sexual activities, and ability to have an erection and health status

	Decline		No decline		OR [95% CI]^c^	*p*
	*n*^a^	% (SE)^b^	*n*^a^	% (SE)^b^		
Sexual desire						
Fair/poor self-rated health	852	25.4 (1.2)	1724	25.1 (0.8)	1.05 [0.84–1.31]	.688
Limiting long-standing illness	852	31.8 (1.3)	1725	29.8 (0.9)	1.13 [0.92–1.38]	.256
Cancer	852	6.5 (0.8)	1725	5.7 (0.5)	1.19 [0.84–1.66]	.327
Coronary heart disease	852	12.1 (0.9)	1725	9.7 (0.7)	1.33 [1.02–1.74]	.038
Stroke	852	4.0 (0.6)	1725	4.0 (0.4)	1.00 [0.66–1.52]	.993
Frequency of sexual activities^d^						
Fair/poor self-rated health	733	20.5 (1.2)	1240	19.9 (0.9)	1.06 [0.82–1.38]	.650
Limiting long-standing illness	733	28.2 (1.3)	1241	24.7 (1.0)	1.23 [0.97–1.54]	.083
Cancer	733	5.4 (0.7)	1241	4.9 (0.6)	1.06 [0.71–1.60]	.771
Coronary heart disease	733	6.9 (0.9)	1241	7.8 (0.7)	0.77 [0.54–1.11]	.156
Stroke	733	2.8 (0.6)	1241	2.9 (0.4)	0.93 [0.53–1.63]	.798
Ability to have an erection						
Fair/poor self-rated health	710	26.4 (1.3)	1858	24.5 (0.8)	1.10 [0.87–1.39]	.440
Limiting long-standing illness	710	31.6 (1.4)	1860	29.9 (0.9)	1.06 [0.85–1.32]	.591
Cancer	710	6.9 (0.8)	1860	5.7 (0.5)	1.18 [0.82–1.68]	.373
Coronary heart disease	710	11.1 (1.1)	1860	10.3 (0.6)	1.07 [0.81–1.42]	.647
Stroke	710	4.1 (0.7)	1860	3.9 (0.4)	1.06 [0.70–1.63]	.777

All percentages and odds ratios are adjusted for age, partnership status, ethnicity, wealth, smoking status, alcohol intake, physical activity and depressive symptoms, and weighted for sampling probabilities and differential nonresponse

^a^Total sample size (unweighted)

^b^Percentage (with standard error) reporting health problem

^c^Adjusted odds ratios (OR) and 95% confidence intervals (CI) for the health outcome of interest in the group reporting a decline in sexual desire/frequency of sexual activities/ability to have an erection relative to the group not reporting a decline

^d^Among those who reported being sexually active

**Table 4:** Prospective associations in men between past-year decline in sexual desire, frequency of sexual activities, and ability to have an erection and incident health problems

	Decline		No decline		OR [95% CI]^c^	*p*
	*n*^a^	% (SE)^b^	*n*^a^	% (SE)^b^		
Sexual desire						
Fair/poor self-rated health	476	14.8 (1.4)	1093	11.7 (0.9)	1.30 [0.94–1.79]	.109
Limiting long-standing illness	430	20.3 (1.6)	1028	15.1 (1.0)	1.41 [1.04–1.91]	.028
Cancer	630	6.7 (0.8)	1320	4.3 (0.6)	1.63 [1.06–2.50]	.026
Coronary heart disease	560	3.6 (0.6)	1277	2.5 (0.4)	1.42 [0.81–2.52]	.225
Stroke	640	3.5 (0.6)	1353	2.7 (0.4)	1.35 [0.78–2.33]	.289
Frequency of sexual activities^d^						
Fair/poor self-rated health	462	14.1 (1.3)	853	10.3 (1.0)	1.47 [1.04–2.08]	.028
Limiting long-standing illness	408	18.0 (1.5)	807	12.1 (1.1)	1.69 [1.20–2.37]	.002
Cancer	575	5.6 (0.8)	985	4.4 (0.6)	1.26 [0.78–2.04]	.341
Coronary heart disease	551	2.8 (0.6)	965	2.8 (0.5)	1.00 [0.53–1.89]	.992
Stroke	587	2.3 (0.5)	1014	2.2 (0.4)	1.04 [0.52–2.11]	.905
Ability to have an erection						
Fair/poor self-rated health	388	16.8 (1.6)	1181	11.3 (0.9)	1.66 [1.19–2.32]	.003
Limiting long-standing illness	354	20.4 (1.8)	1103	15.4 (1.0)	1.34 (0.97–1.87]	.077
Cancer	516	7.1 (0.9)	1433	4.4 (0.5)	1.73 [1.11–2.70]	.015
Coronary heart disease	488	5.0 (0.7)	1376	2.1 (0.4)	2.29 [1.29–4.07]	.005
Stroke	525	3.4 (0.7)	1466	2.8 (0.4)	1.37 [0.78–2.40]	.270

All percentages and odds ratios are adjusted for age, partnership status, ethnicity, wealth, smoking status, alcohol intake, physical activity and depressive symptoms, and weighted for sampling probabilities and differential nonresponse

^a^Total sample size (unweighted)

^b^Percentage (with standard error) reporting incident health problem

^c^Adjusted odds ratios (OR) and 95% confidence intervals (CI) for the health outcome of interest in the group reporting a decline in sexual desire/frequency of sexual activities/ability to have an erection relative to the group not reporting a decline

^d^Among those who reported being sexually active

**Table 5:** Cross-sectional associations in women between past-year decline in sexual desire, frequency of sexual activities, and ability to become sexually aroused and health status

	Decline		No decline		OR [95% CI]^c^	*p*
	*n*^a^	% (SE)^b^	*n*^a^	% (SE)^b^		
Sexual desire						
Fair/poor self-rated health	1010	25.1 (1.2)	2185	25.5 (0.8)	0.89 [0.72–1.09]	.248
Limiting long-standing illness	1009	36.5 (1.3)	2185	35.0 (0.9)	1.09 (0.91–1.31)	.367
Cancer	1010	5.6 (0.7)	2185	5.8 (0.5)	0.91 [0.65–1.29]	.612
Coronary heart disease	1010	7.8 (0.7)	2185	6.5 (0.5)	1.14 [0.83–1.56]	.420
Stroke	1010	3.9 (0.5)	2185	3.1 (0.4)	1.17 [0.77–1.79]	.457
Frequency of sexual activities^d^						
Fair/poor self-rated health	666	19.5 (1.3)	1074	18.8 (1.0)	1.09 [0.82–1.46]	.555
Limiting long-standing illness	665	30.1 (1.5)	1074	27.3 (1.2)	1.29 [1.01–1.65]	.040
Cancer	666	6.0 (0.9)	1074	5.5 (0.7)	1.01 [0.65–1.57]	.982
Coronary heart disease	666	5.3 (0.7)	1074	3.5 (0.6)	1.86 [1.07–3.24]	.029
Stroke	666	3.4 (0.6)	1074	1.7 (0.4)	2.04 [1.03–4.03]	.041
Ability to become sexually aroused^d^						
Fair/poor self-rated health	362	20.7 (1.7)	1005	16.9 (1.0)	1.37 [0.95–1.98]	.090
Limiting long-standing illness	362	27.6 (2.0)	1005	27.1 (1.2)	1.09 [0.80–1.48]	.587
Cancer	362	6.4 (1.1)	1005	5.1 (0.7)	1.27 [0.74–2.18]	.395
Coronary heart disease	362	4.2 (0.9)	1005	3.5 (0.5)	1.51 [0.75–3.06]	.249
Stroke	362	3.6 (0.7)	1005	1.6 (0.4)	2.36 [1.04–5.35]	.040

All percentages and odds ratios are adjusted for age, partnership status, ethnicity, wealth, smoking status, alcohol intake, physical activity and depressive symptoms, and weighted for sampling probabilities and differential nonresponse

^a^Total sample size (unweighted)

^b^Percentage (with standard error) reporting incident health problem

^c^Adjusted odds ratios (OR) and 95% confidence intervals (CI) for the health outcome of interest in the group reporting a decline in sexual desire/frequency of sexual activities/ability to have an erection relative to the group not reporting a decline

^d^Among those who reported being sexually active

**Table 6:** Prospective associations in women between past-year decline in sexual desire, frequency of sexual activities, and ability to become sexually aroused and incident health problems

	Decline		No decline		OR [95% CI]^c^	*p*
	*n*^a^	% (SE)^b^	*n*^a^	% (SE)^b^		
Sexual desire						
Fair/poor self-rated health	581	16.3 (1.4)	1395	12.9 (0.9)	1.26 [0.93–1.70]	.141
Limiting long-standing illness	485	15.7 (1.6)	1227	16.5 (1.0)	0.89 [0.64–1.22]	.460
Cancer	762	4.4 (0.8)	1720	6.2 (0.5)	0.67 [0.44–1.03]	.069
Coronary heart disease	732	2.0 (0.5)	1720	1.8 (0.3)	1.00 [0.52–1.95]	.995
Stroke	760	2.4 (0.5)	1764	2.3 (0.3)	1.34 [0.72–2.47]	.356
Frequency of sexual activities^d^						
Fair/poor self-rated health	429	13.0 (1.4)	756	8.7 (1.0)	1.64 [1.07–2.51]	.022
Limiting long-standing illness	362	12.3 (1.7)	671	12.6 (1.2)	0.93 [0.60–1.42]	.721
Cancer	513	3.7 (0.9)	872	6.0 (0.7)	0.60 [0.34–1.07]	.081
Coronary heart disease	506	1.7 (0.5)	892	1.1 (0.4)	1.52 [0.56–4.13]	.415
Stroke	519	1.3 (0.5)	901	1.2 (0.4)	1.22 [0.41–3.67]	.719
Ability to become sexually aroused^d^						
Fair/poor self-rated health	242	9.7 (1.8)	712	8.8 (1.0)	1.12 [0.64–1.95]	.686
Limiting long-standing illness	211	12.1 (2.1)	622	11.7 (1.2)	0.97 [0.56–1.67]	.906
Cancer	284	4.1 (1.3)	814	5.2 (0.7)	0.76 [0.38–1.55]	.451
Coronary heart disease	281	1.4 (0.6)	829	1.1 (0.4)	0.84 [0.23–3.14]	.797
Stroke	285	1.2 (0.6)	842	1.0 (0.3)	1.06 [0.24–4.63]	.943

All percentages and odds ratios are adjusted for age, partnership status, ethnicity, wealth, smoking status, alcohol intake, physical activity and depressive symptoms, and weighted for sampling probabilities and differential nonresponse

^a^Total sample size (unweighted)

^b^Percentage (with standard error) reporting incident health problem

^c^Adjusted odds ratios (OR) and 95% confidence intervals (CI) for the health outcome of interest in the group reporting a decline in sexual desire/frequency of sexual activities/ability to have an erection relative to the group not reporting a decline

^d^Among those who reported being sexually active

Among men, those who reported a decline in sexual desire had 33% higher odds of reporting CHD at baseline than those who reported stable or increased sexual desire (12.1% vs. 9.7%, 95% CI 1.02–1.74, *p* = .038) (Table 3). There were no other significant cross-sectional associations in men. Prospectively, men who reported a decline in sexual desire had 41% higher odds of incident limiting long-standing illness (20.3% vs. 15.1%, 95% CI 1.04–1.91, *p* = .028) and 63% higher odds of incident cancer (6.7% vs. 4.3%, 95% CI 1.06–2.50, *p* = .026) over four-year follow-up than those who did not (Table 4). Men who reported a decline in the frequency of sexual activities had 47% higher odds of deterioration in self-rated health (14.1% vs. 10.3%, 95% CI 1.04–2.08, *p* = .028) and 69% higher odds of incident limiting long-standing illness (18.0% vs. 12.1%, 95% CI 1.20–2.37, *p* = .002) than those who did not. Men who reported a decline in their ability to have an erection had 66% higher odds of deterioration in self-rated health (16.8% vs. 11.3%, 95% CI 1.19–2.32, *p* = .003), 73% higher odds of incident cancer (7.1% vs. 4.4%, 95% CI 1.11–2.70, *p* = .015) and 129% higher odds of incident CHD (5.0% vs. 2.1%, 95% CI 1.29–4.07, *p* = .005) than those who did not.

Among women, those who reported a decline in the frequency of sexual activities had 29% higher odds of reporting limiting long-standing illness (30.1% vs. 27.3%, 95% CI 1.01–1.65, *p* = .040), 86% higher odds of CHD (5.3% vs. 3.5%, 95% CI 1.07–3.24, *p* = .029) and 104% higher odds of stroke (3.4% vs. 1.7%, 95% CI 1.03–4.03, *p* = .041) at baseline than those who did not (Table 5). In addition, those who reported a decline in their ability to become sexually aroused had 136% higher odds of reporting a stroke (3.6% vs. 1.6%, 95% CI 1.04–5.35, *p* = .040) at baseline than those who did not. The only significant prospective association was between a decline in frequency of sexual activities and deterioration of self-rated health, with women who reported a decline having 64% higher odds of incident fair/poor self-rated health not (13.0% vs. 8.7%, 95% CI 1.07–2.51, *p* = .022) than those who did not (Table 6).

Imputing missing values did not substantially alter the pattern of results, although some associations were attenuated (Supplementary Tables 1 and 2). In men, associations that were weaker when missing values were imputed were the prospective associations between sexual desire and limiting long-standing illness (from OR 1.41 to OR 1.31) and cancer (from OR 1.63 to OR 1.29), frequency of sexual activities and self-rated health (from OR 1.47 to OR 1.30) and limiting long-standing illness (from OR 1.69 to OR 1.54), and ability to have an erection and cancer (from OR 1.73 to OR 1.49). In women, associations that were weaker when missing values were imputed were the cross-sectional associations between frequency of sexual activities and CHD (from OR 1.86 to OR 1.47) and stroke (from OR 2.04 to OR 1.46), and ability to become sexually aroused and stroke (from OR 2.36 to OR 2.07), and the prospective association between frequency of sexual activities and self-rated health (from OR 1.64 to OR 1.38).

## Discussion

In a large, representative sample of English older adults, past-year decline in sexuality was associated with problems in a range of health outcomes in males and females. One critical finding was that a decline in frequency of sexual activities was longitudinally associated with deterioration in self-rated health for both men and women. Other associations between sexuality and health outcomes were primarily longitudinal in nature for men and cross sectional for women. For men, a decline in sexual desire was associated with higher odds of reporting CHD at baseline and higher odds of incident limiting long-standing illness and incident cancer over four-year follow-up. In addition, a decline in frequency of sexual activity was longitudinally associated with higher odds of incident limiting long-standing illness. In men, those who reported a decline in their ability to have an erection had higher odds of deterioration in self-rated health, incident cancer, and CHD. In women, cross-sectional associations were found between a decline in frequency of sexual activities and higher odds of reporting limiting long-standing illness and higher odds of stroke, and a decline in ability to become sexually aroused was associated with higher odds of reporting stroke.

These findings support those of previous studies that were smaller or cross sectional or theoretical in design (Bosland, 1988; Brody & Preut, 2003; Ebrahim et al., 2002; Flynn & Gow, 2015; Le et al., 1989). Emphasis should be placed on the findings from the longitudinal analyses, specifically that a decline in sexual activity or desire was associated with limiting long-standing illness, self-rated health, and incident cancer at four-year follow-up, predominantly in men. Several mechanisms may explain the observed associations. Firstly, during sexual activity or at the time sexual intercourse is at its peak, there is a release of endorphins, endogenous opioid peptides that function as neurotransmitters, which generates a happy or blissful feeling (Rokade, 2011). Importantly, circulating endorphin levels have been shown to be associated with higher natural killer cell activity (Darko et al., 1992). A higher natural killer cell activity may be associated with a lower risk of cancer and viruses; they have also been found to prevent against infections of the lungs and play an important role in improving asthma and many other conditions (Mandal & Viswanathan, 2015; Wu & Lanier, 2003) and thus are also likely to be related to limiting long-standing illness and self-rated health. However, it should be noted here that endorphins are most likely to be released when sexual activity is at its peak (i.e., during orgasm). The present study did not collate information pertaining to orgasms. Second, sex can be considered a form of physical activity; one study conducted in a younger population (22.6 ± 2.8 years) showed that energy expenditure during sexual activity was 85 kCal or 3.6 kCal/min and was performed at a moderate intensity (5.8 METS) (Frappier et al., 2013). Therefore, it is possible that one may acquire health benefits from regular participation in physical activity via sexual activity. Physical activity has been shown to be associated with better self-rated health, lower odds of having a limiting long-standing illness, and lower incidence of certain cancers (McPhee et al., 2016; Sodergren et al., 2008). Third, it is possible that early symptoms of cancer and long-standing illness may predict a decline in sexual activity and desire before diagnosis of the conditions. For example, fatigue is a reported early warning sign for cancer and is often experienced before a diagnosis is made (de Nooijer, Lechner, & de Vries, 2001); it is plausible to assume that fatigue will be associated with a reduction in sexual activity. It is likely that the observed associations between declines in sexual activity and desire with the varying health outcomes result from a combination of all the discussed pathways. The fact that associations were predominantly cross sectional in women and longitudinal in men suggests that there may be more reverse causation in women. It is possible that once women acquire a physical illness, they are more likely to become asexual because their level of desire is lower to begin with (Lee et al., 2016).

The finding that erectile dysfunction is associated with self-rated health, incident cancer, and CHD is one that is important and supports and adds to previous literature. Indeed, a recent narrative review concluded that patients with erectile dysfunction are at an increased risk of CHD morbidity and/or mortality as well as for all-cause mortality (Katsiki, Wierzbicki, & Mikhailidis, 2015). This may be explained by the fact that vascular factors are a risk factor for erectile dysfunction as well as CHD. A recent meta-analysis also reported a high prevalence of erectile dysfunction among men with diabetes (Kouidrat et al., 2017). As is already standard practice for many clinicians, these findings highlight the importance of monitoring male patients with erectile dysfunction by assessing their vascular risk and preventing or adequately treating CHD risk factors (Katsiki et al., 2015).

To our knowledge, this is the first study to suggest that erectile dysfunction may be associated with the onset of cancer. It is possible that this association may be explained by certain risk factors associated with both cancer and erectile dysfunction, such as obesity, a high-fat diet, smoking, or alcohol use. However, as the present analyses controlled for smoking and alcohol use, it is unlikely that these confounding variables are driving the observed association. An alternative explanation is that erectile difficulties may be caused by psychological factors relating to pre-diagnostic cancer symptoms, for example, stress, anxiety, or depression (Shabsigh et al., 1998), although the latter was controlled for in the present analyses by entering depression into our statistical models as a covariate. Further research is required to test these suggested pathways.

Interestingly, in women a decline in the ability to become sexually aroused was cross-sectionally associated with higher odds of having had a stroke. While the direction of causation cannot be established from these cross-sectional data, given that we did not observe a longitudinal association from decline in sexual arousal to increased odds of stroke, it is possible that reduced sexual arousal may be a consequence of stroke. Literature shows that in women the first sign of sexual arousal is an increase in the blood flow to the vaginal wall (Levin, 1991). Diminished pelvic blood flow secondary to aortoiliac or atherosclerotic disease leads to vaginal wall and clitoral smooth muscle fibrosis. This can ultimately result in symptoms of vaginal dryness and dyspareunia (Berman, 2005), which may result in a decrease in sexual desire. It is also plausible that brain areas involved in sexual desire (e.g., the amygdala) may be impaired in those who have a stroke. Atherosclerotic disease may also lead to an increased risk of cerebrovascular disease (Blaton, 2004). Another possible explanation for this finding is that difficulty becoming sexually aroused may cause decline in sex-related physical activity, which may help protect against atherosclerosis and cerebrovascular disease (Bowles & Laughlin, 2011; Kronenberg et al., 2000).

This is the first study to investigate the cross-sectional and longitudinal associations between decline in sexuality (sexual desire, frequency of sexual activity, and sexual function) and health problems in a large representative sample of older adults. Strengths of the study include the large representative sample of English older adults and adjustment for a range of sociodemographic and health-related confounders. However, there were also several limitations. First, despite the longitudinal design, it is not known whether changes in sexual activity, desire, and function play an etiological role in disease, or whether early stages of disease onset lead to a reduction in sexual activity and desire. It is likely to be a combination of both. Further research using longer follow-ups or an experimental design to avoid lead time bias is required to explore the direction of causation and may detect additional associations that it was not possible to pick up with a four-year follow-up period. Secondly, participants may have already had undiagnosed disease at baseline, which might have had an influence on their sexual behavior. For example, it could be the case that participants with undiagnosed CHD were avoiding sexual activity because it exacerbated their symptoms (e.g., shortness of breath, chest pain). Thirdly, sexual information was self-reported, and people may not respond honestly to questions for fear of being judged. However, participants were informed that survey responses would remain anonymous. Moreover, there is currently little other option to measure the exposure variables investigated in the present study other than by self-report. Information on the health outcomes was also self-reported potentially introducing reporting bias. The wide time frame used to capture changes in sexuality (a period of a year) may preclude identification of more acute changes that relate to the onset of health conditions. Fourthly, while we adjusted for a range of socio-demographic and health-related covariates measured at baseline, it is possible that the status of these covariates may have changed over the course of the study period. In addition, data on partner’s health were not included; future studies may wish to explore the influence of ill health in the partner on the relationship between a decline in sexuality and health. Our measure of sexual activity was relatively broad including intercourse, masturbation and petting/fondling. Relationships between each individual component and health outcomes and their relative contribution to effects observed in relation to each health outcome may vary. Fifthly, a substantial number of ELSA participants were excluded on the basis of missing data. While imputing missing values produced results similar to those observed in the complete cases, some associations were attenuated. Finally, the prospective sample was younger, wealthier, and healthier than those for whom follow-up data were not available (Online Appendix 1), in line with retention in other longitudinal studies (Mendes de Leon, 2007). Given the lower prevalence of disease in the prospective subsample, it is possible that longitudinal associations were underestimated. With many of the outcomes occurring infrequently (e.g., cancer, stroke), we lacked sufficient power to detect significant effects.

With our results indicating that changes in sexual activity, desire, and function may predict the onset of cancer and CHD in older adults, questions are raised about the potential to screen for such changes in older age in order to identify individuals at increased risk for developing these conditions. Research indicates that although general practitioners recognize that sexuality may remain an important concern in later life, they do not proactively discuss sexual health with older patients (Gott, Hinchliff, & Galena, 2004). Reasons for this include a perception that sexual health issues are not relevant to older people; feeling that asking about sex may be an invasion of privacy and offend older patients, leading to breakdown of the doctor/patient relationship; and feeling uncomfortable discussing sexual health with older patients due to lack of training and preconceived ideas about sexuality in older age (Gott et al., 2004). Whether or not screening for changes in sexual activity/desire in older patients is acceptable and amenable to change should be explored. Another area for future research is investigation into whether increases in sexual activity and function are associated with reduced risk of adverse health outcomes. We were unable to explore this in the present study due to the very small number of participants reporting increases; for example, just 143 men and 89 women reported an increase in sexual desire over the past year.

### Conclusions

The present study revealed novel associations between declines in sexual activity and function and a range of health outcomes. To varying degrees in men and women, reductions in sexual activity and desire and increases in sexual problems were associated with greater risk of cancer, CHD, stroke, and, more broadly, limiting long-standing illness and poor self-rated health. These findings have important implications for research and clinical practice. Physicians should be mindful that older adults are not asexual and that a decline in their sexual activity or desire and the onset of sexual problems may indicate ill health. The development of interventions to promote sexual health and well-being at older ages may offer considerable opportunities to reduce the burden of disease in later life.

## Electronic supplementary material

Below is the link to the electronic supplementary material.

Supplementary material 1 (DOCX 18 kb)
