# Supplementary material for: Declines in Sexual Activity and Function Predict Incident Health Problems in Older Adults: Prospective Findings from the English Longitudinal Study of Ageing
Source: Arch Sex Behav. 2019 Aug 20;49(3):929–40. doi: 10.1007/s10508-019-1443-4 (PMC7058559; doi:10.1007/s10508-019-1443-4)
Supplement: Supplementary file 1 — Supplementary material 1 (DOCX 18 kb) [file 10508_2019_1443_MOESM1_ESM.docx]

| **Supplementary Table 1** Cross-sectional and prospective associations in men between past-year decline in sexual desire, frequency of sexual activities, and ability to become sexually aroused and health problems: missing values imputed | | | | | | | | |  |  |
| --- | --- | --- | --- | --- | --- | --- | --- | --- | --- | --- |
|  |  | **Cross-sectional** | | |  | | **Prospective** | | | |
|  |  | **OR [95% CI]^1^** | ***p*** |  | | **OR [95% CI]^1^** | | ***p*** | |  |
| **Sexual desire** | |  |  |  | |  | |  | |  |
|  | Fair/poor self-rated health | 1.05 [0.84-1.31] | .664 |  | | 1.24 [0.89-1.73] | | .209 | |  |
|  | Limiting long-standing illness | 1.13 [0.92-1.39] | .239 |  | | 1.31 [0.97-1.77] | | .078 | |  |
|  | Cancer | 1.19 [0.85-1.67] | .325 |  | | 1.29 [0.77-2.17] | | .315 | |  |
|  | Coronary heart disease | 1.33 [1.02-1.74] | .037 |  | | 1.39 [0.82-2.35] | | .218 | |  |
|  | Stroke | 1.00 [0.66-1.52] | .992 |  | | 1.29 [0.55-3.01] | | .528 | |  |
| **Frequency of sexual activities^2^** | |  |  |  | |  | |  | |  |
|  | Fair/poor self-rated health | 1.10 [0.87-1.38] | .422 |  | | 1.30 [0.84-2.01] | | .216 | |  |
|  | Limiting long-standing illness | 1.22 [0.91-1.66] | .175 |  | | 1.54 [1.04-2.28] | | .033 | |  |
|  | Cancer | 1.13 [0.77-1.66] | .518 |  | | 1.07 [0.67-1.71] | | .765 | |  |
|  | Coronary heart disease | 0.98 [0.66-1.45] | .895 |  | | 0.94 [0.50-1.76] | | .841 | |  |
|  | Stroke | 1.03 [0.52-2.04] | .932 |  | | 1.20 [0.48-2.98] | | .664 | |  |
| **Ability to have an erection** | |  |  |  | |  | |  | |  |
|  | Fair/poor self-rated health | 1.11 [0.87-1.40] | .401 |  | | 1.64 [1.21-2.23] | | .002 | |  |
|  | Limiting long-standing illness | 1.08 [0.87-1.34] | .505 |  | | 1.32 [0.97-1.80] | | .074 | |  |
|  | Cancer | 1.17 [0.82-1.66] | .401 |  | | 1.49 [0.93-2.37] | | .094 | |  |
|  | Coronary heart disease | 1.08 [0.81-1.44] | .598 |  | | 2.26 [1.24-4.09] | | .008 | |  |
|  | Stroke | 1.01 [0.65-1.55] | .973 |  | | 1.15 [0.61-2.19] | | .652 | |  |
| ^1^ Pooled adjusted odds ratios (OR) and 95% confidence intervals (CI) for the health outcome of interest in the group reporting a decline in sexual desire/frequency of sexual activities/ability to become sexually aroused relative to the group not reporting a decline.  ^2^ Among those who reported being sexually active.  All percentages and odds ratios are adjusted for age, partnership status, ethnicity, wealth, smoking status, alcohol intake, physical activity and depressive symptoms, and weighted for sampling probabilities and differential nonresponse. | | | | | | | | |  |  |

| **Supplementary Table 2** Cross-sectional and prospective associations in women between past-year decline in sexual desire, frequency of sexual activities, and ability to become sexually aroused and health problems: missing values imputed | | | | | | | | |  |  |
| --- | --- | --- | --- | --- | --- | --- | --- | --- | --- | --- |
|  |  | **Cross-sectional** | | |  | | **Prospective** | | | |
|  |  | **OR [95% CI]^1^** | ***p*** |  | | **OR [95% CI]^1^** | | ***p*** | |  |
| **Sexual desire** | |  |  |  | |  | |  | |  |
|  | Fair/poor self-rated health | 0.89 [0.73-1.07] | .257 |  | | 1.24 [0.87-1.78] | | .228 | |  |
|  | Limiting long-standing illness | 1.09 [0.99-1.20] | .361 |  | | 0.91 [0.65-1.28] | | .585 | |  |
|  | Cancer | 0.90 [0.76-1.08] | .567 |  | | 0.76 [0.49-1.20] | | .235 | |  |
|  | Coronary heart disease | 1.14 [0.97-1.34] | .409 |  | | 1.07 [0.57-1.98] | | .839 | |  |
|  | Stroke | 1.18 [0.95-1.47] | .436 |  | | 1.15 [0.65-2.04] | | .625 | |  |
| **Frequency of sexual activities^2^** | |  |  |  | |  | |  | |  |
|  | Fair/poor self-rated health | 1.05 [0.78-1.42] | .715 |  | | 1.38 [0.93-2.04] | | .104 | |  |
|  | Limiting long-standing illness | 1.25 [1.04-1.51] | .017 |  | | 1.12 [0.83-1.52] | | .441 | |  |
|  | Cancer | 0.97 [0.66-1.41] | .850 |  | | 0.76 [0.44-1.32] | | .302 | |  |
|  | Coronary heart disease | 1.47 [0.90-2.40] | .119 |  | | 1.15 [0.64-2.05] | | .641 | |  |
|  | Stroke | 1.46 [0.95-2.26] | .084 |  | | 0.93 [0.56-1.53] | | .773 | |  |
| **Ability to become sexually aroused^2^** | |  |  |  | |  | |  | |  |
|  | Fair/poor self-rated health | 1.02 [0.79-1.32] | .877 |  | | 1.39 [0.93-2.09] | | .105 | |  |
|  | Limiting long-standing illness | 1.06 [0.86-1.32] | .574 |  | | 1.19 [0.73-1.95] | | .442 | |  |
|  | Cancer | 1.13 [0.78-1.63] | .528 |  | | 0.88 [0.29-2.61] | | .776 | |  |
|  | Coronary heart disease | 1.35 [0.76-2.38] | .276 |  | | 1.12 [0.45-2.75] | | .795 | |  |
|  | Stroke | 2.07 [0.91-4.70] | .077 |  | | 1.69 [0.75-3.77] | | .183 | |  |
| ^1^ Pooled adjusted odds ratios (OR) and 95% confidence intervals (CI) for the health outcome of interest in the group reporting a decline in sexual desire/frequency of sexual activities/ability to become sexually aroused relative to the group not reporting a decline.  ^2^ Among those who reported being sexually active.  All percentages and odds ratios are adjusted for age, partnership status, ethnicity, wealth, smoking status, alcohol intake, physical activity and depressive symptoms, and weighted for sampling probabilities and differential nonresponse. | | | | | | | | |  |  |
